# Burden of peripheral arterial disease in Europe and the United States: a patient survey

**DOI:** 10.1186/1477-7525-11-175

**Published:** 2013-10-22

**Authors:** Elizabeth Marrett, Marco daCosta DiBonaventura, Qiaoyi Zhang

**Affiliations:** 1 Global Health Outcomes, Merck Sharp & Dohme Corp, Whitehouse Station, NJ, USA; 2 Health Outcomes Practice, Kantar Health, 11 Madison Avenue, 12th Floor, New York, NY 10010, USA

**Keywords:** Peripheral arterial disease, Health-related quality of life, Work productivity and activity impairment, Healthcare resource use

## Abstract

**Background:**

The aim of the current study was to quantify the burden of peripheral arterial disease (PAD) with respect to health-related quality of life, work productivity and activity impairment, and healthcare resource utilization.

**Methods:**

Data were obtained from the 2010 EU National Health and Wellness Survey (NHWS), which included participants from France, Germany, Italy, Spain, and the UK (5EU, N = 57,805) as well as the 2010 US NHWS (N = 75,000). The NHWS is an annual, cross-sectional, self-administered Internet survey which employs a stratified random sampling frame to match the age and gender characteristics of the NHWS sample with known population statistics. Participants who self-reported a diagnosis of PAD were compared with participants who did not self-report a diagnosis of PAD on health-related quality of life (mental and physical component summary scores and health utilities from the Short Form-12v2), work productivity and activity impairment (Work Productivity and Activity Impairment questionnaire), and healthcare resource use in terms of the number of physician visits, emergency room visits, and hospitalizations in the past six months through regression modeling adjusting for demographics and health characteristics.

**Results:**

A total of 743 (1.29%) and 777 (1.04%) participants self-reported a diagnosis of PAD in the 5EU and US, respectively. After adjusting for demographics and health characteristics, patients with PAD reported worse health-related quality of life, as measured by health utilities (5EU: 0.66 vs. 0.70; US: 0.66 vs. 0.72; all p < .05), greater overall work impairment percentage (5EU: 38.27% vs. 27.48%; US: 23.89% vs. 14.26%) and greater healthcare resource use compared to participants without PAD (all p < .05).

**Conclusions:**

These results suggest a significant burden for patients with PAD in both the 5 EU countries and the US with respect to both quality of life and economic outcomes. Improved management of these patients may have profound effects from both patient and societal perspectives.

## Background

Peripheral arterial disease (PAD) is a condition which results from atherosclerosis in the abdomen or lower extremities [1]. Pain in the muscles of the legs during walking, known as intermittent claudication, is the most common clinical presentation [2]. Left untreated, PAD can progress to chronic pain in the legs and eventually to non-healing wounds, gangrene, and limb loss [3]. Morbidity and mortality risk among PAD patients is high. Caro and colleagues (2005) examined 16,440 patients in Canada and found that within an average of 6 years following diagnosis, 9.7% of patients had a stroke, 9.5% had a myocardial infarction, and 48.5% had died [4]. Although it is frequently undiagnosed [1], it has been estimated that 4.3% of the United States (US) population 40 years and older has PAD [5]. A similar prevalence of PAD has been found in Western Europe, with population studies estimating the prevalence between 4–8% [6-9].

Predominantly due to the functional limitations caused by claudication [10,11], patients with PAD report significantly lower health-related quality of life compared with the general population on every Short Form-36 (SF-36) domain [12]. Similarly, Leicht et al. (2010) reported physical SF-36 scores (physical component summary (PCS) = 36.9) more than 1 standard deviation below the population norm of 50 [13].

Aside from the impact on patient health-related quality of life, PAD also poses an economic burden from a societal perspective. In the US, PAD was associated with an increase in hospitalizations by 0.32 times per year [14]. Indeed, PAD-related hospitalizations, most frequently due to either stroke or MI, were the largest contributors of PAD-related costs per patient (nearly 75% of total costs), which summed to nearly $6,000 per patient per year [14]. More recent estimates have found similar vascular-related hospitalization costs, with per-patient biennial costs ranging from $7,000 for patients with a history of claudication to $10,430 for patients with revascularization [15]. Costs in the European Union (EU) are also high. Two-year vascular-related hospitalization costs for patients with PAD were €3182 in France and €2724 in Germany [16]. Because of the functional limitations associated with claudication and ischemia, it is generally thought that PAD is also associated with significant work loss [17]. However, the extent of this relationship is not well known.

The objective of the current study was to further this line of research to better understand the burden that PAD poses from both a health-related quality of life and economic perspective. Although health-related quality of life and health utility values are often used as endpoints in studies assessing PAD treatments [18-21] or as a predictor or associated factor of physical functioning [10,11,13,22,23], less is known about the effect of PAD on the health-related quality of life of general PAD samples in Europe or the US. Similarly, very little is known about the effect of PAD on rates of work productivity loss. The generalizability of the existing studies is also questionable since few, if any, attempts were made to recruit samples from a broad population. The aim of the current study was to address these gaps by investigating the association between PAD and health-related quality of life, work productivity loss, and healthcare resource use, using a large, nationally-representative (with respect to age and gender) data source for both the 5EU (i.e., France, Germany, Italy, Spain, and the UK) and the US.

## Methods

### Data source

Data were obtained from the 2010 5EU National Health and Wellness survey (NHWS), which included participants from France, Germany, Italy, Spain, and the UK (N = 57,805), as well as the 2010 US NHWS (N = 75,000). The NHWS is an annual, cross-sectional, self-administered Internet survey administered to a sample of adults who were identified through a web-based consumer panel (i.e., a pre-recruited sample of adults who agree to participate in survey research including, but not limited to, the NHWS). Members of the panel are recruited through opt-in emails, co-registration with panel partners, e-newsletter campaigns, and online banner placements. All panel members (over two million in the US and 5EU) explicitly agreed to join the panel, registered through unique email addresses, and completed in-depth demographic registration profiles. The NHWS is just one of many potential surveys that any given panel member might be invited to complete.

In all countries, a stratified random sampling frame was implemented to ensure that the distribution of age, gender and ethnicity (US only) of the NHWS sample matched that of the respective countries according to either the International Database of the Census (5EU) or the US Census (United States Census Bureau, 2011). More specifically, those governmental sources were used to obtain the proportions of various demographic groups (e.g., the proportion of the total adult population which was 18–29 years old and male) among the adult population of each country. All panel members were then categorized into these demographic subgroups and were randomly sampled to complete the NHWS in such a way that the above mentioned demographic characteristics of the final NHWS matched those of each country’s population. All participants from 5EU (N = 57,805) and US (N = 75,000) NHWS databases were included in the analyses.

Participants who completed the NHWS received compensation in the form of points, which could be redeemed for small prizes or entered into a drawing. All participants provided informed consent and the study was approved by Essex Institutional Review Board, Inc (Lebanon, NJ, USA; 5EU Approval reference number: KH-NHWS-EU2010; US Approval reference number: CHS-NHWS-US2010-21105). Additional details on the sampling methods are provided elsewhere [24,25].

### Measures

#### Independent variable

##### PAD diagnosis

All participants in the NHWS were asked whether they had ever been diagnosed with PAD by a physician. Those who responded affirmatively were considered to have PAD while all others were considered not to have PAD. No other inclusion or exclusion criteria were applied.

#### Outcome measures

##### Health-related quality of life

The Medical Outcomes Study 12-Item Short Form Survey Instrument (SF-12v2), a multipurpose, generic health-related quality of life instrument comprised of 12 questions [26], was administered in the survey. The SF-12v2 items are used to generate two health-related quality of life summary scores: the physical component summary (PCS) and mental component summary (MCS) scores, each of which are normed to the population (a mean of 50 and a standard deviation of 10). The items from the SF-12v2 were also used to generate a health state utility (using the Short Form-6D, or SF-6D, classification). The SF-6D classification is a method to calculate a preference-based single index for health using general population values, which varies (conceptually) from 0 to 1 with higher scores indicating a better health state [27]. As is standard for applying the SF-6D classification, UK weights were applied to create health utility values.

##### Work productivity

Work productivity was assessed using the Work Productivity and Activity Impairment-General Health (WPAI-GH; http://www.reillyassociates.net/WPAI_General.html) questionnaire, a 6-item validated instrument which consists of four metrics: absenteeism (the percentage of work time missed because of one’s health in the past seven days), presenteeism (the percentage of impairment experienced while at work in the past seven days because of one’s health), overall work productivity loss (an overall impairment estimate that is a combination of absenteeism and presenteeism), and activity impairment (the percentage of impairment in daily activities because of one’s health in the past seven days) [28]. Only participants who reported being employed full-time or part-time provided data for absenteeism, presenteeism, and overall work impairment percentages. All participants provided data for activity impairment.

##### Healthcare resource use

Healthcare resource use was defined by the number of healthcare provider visits, the number of emergency room (ER) visits, and the number of times hospitalized in the past six months. All measures were reported by the participant. Resource use events were from all causes and were not specific to PAD.

#### Covariates

##### Demographics and health characteristics

Baseline differences between those with and without PAD were examined for age, gender (male or female), ethnicity (for US analyses only; non-Hispanic white, non-Hispanic black, Hispanic, or other), education (university degree vs. less than university degree), household income (5EU analyses: below country-specific median, above country-specific median, or decline to answer; US analyses: <$25 K, $25 K to < $50 K, $50 K to <$75 K, $75 K or more, or decline to answer), alcohol use (currently drink vs. do not currently drink), exercise behavior (currently exercise vs. do not currently exercise) and comorbidities (defined as the presence or absence of the following comorbidity clusters, as described in Additional file 1: cancer, gastrointestinal disease, infectious disease, arthritis, psychiatric disease, respiratory disease, renal disease, liver disease, diabetes, cardiovascular disease, and cerebrovascular disease). Comorbidities were also assessed using the Charlson comorbidity index (CCI) which is a single index score measuring the overall comorbidity burden of a patient [29]. Participants were also asked to provide their height and weight which was then used to calculate body mass index (BMI; underweight, normal, overweight, obese, or decline to provide weight).

#### Analyses

All analyses were conducted separately for the 5EU and US. Descriptive statistics were used to summarize participant demographics, health behaviors, comorbid conditions and patient reported outcomes. Differences in these factors between participants with and without PAD (using all participants from each respective NHWS) were assessed using t-tests for continuous variables and chi-square tests for categorical variables. Due to a large imbalance with respect to mean age between disease groups, we also conducted a 1-to-1 hard match to minimize potential bias with respect to measured outcomes. As such, each participant with PAD was randomly matched to a single respondent of the same age without PAD from the full survey cohort, and similar analyses were repeated based on this sample.

Regression modeling was conducted to assess adjusted differences in least square means between participants with PAD and the age-matched group without PAD for all patient reported outcomes. MCS, PCS, and health utilities were examined using a general linear model; WPAI scores and resource use variables were assessed using a generalized linear model (specifying a log-link function and a negative binomial distribution due to overdispersion). All regression analyses included the following covariates: gender, education level, BMI, smoking, drinking and exercise status, and comorbid conditions.

Health utilities were also assessed by individual 5EU countries. Unlike the overall 5EU analyses, 1:1 hard matching on age was not performed at the individual country level due to challenges associated with smaller sample sizes. However, age was added as a covariate in the regression models.

For all regression models (these are hereafter referred to “adjusted analyses”), adjusted means were reported. Adjusted means represent the predicted value for the outcome (separately for participants with and without PAD), assuming all covariates are set at the sample mean. For the general linear models, adjusted means were calculated using a least-squares algorithm; for the generalized linear models, adjusted means were calculated using a maximum likelihood algorithm and exponentiated to readjust the means to the original metric (rather than the log of the original metric).

## Results

A total of 743 participants (1.29%) in the 5EU, and 777 participants (1.04%) in the US self-reported a diagnosis of PAD. Overall, demographics and health histories differed between these participants and those not reporting a diagnosis of PAD (Table 1). In both the 5EU and US, those diagnosed with PAD were significantly older and less likely to have a university or college degree, have an above median income, be currently employed, currently consume alcohol, and to regularly exercise (all p < .05). These patients were also significantly more likely to be obese, to currently smoke and they had a greater comorbidity burden as indicated by the CCI (all p < .05). In addition, all comorbidity clusters were significantly more prevalent among those with PAD than those without PAD in both regions (all p < .05). The only observed difference between the 5EU and the US was with regard to gender. In the 5EU, there was no difference between the proportion of males with and without PAD (47.2% vs. 48.6%). However, in the US there were significantly more males with PAD than without the disease (59.5% vs. 48.1%, p < .05).

**Table 1:** Demographic, health characteristic, and comorbidity differences between those with and without PAD in the 5EU and United States

	**5EU**		**US**	
	**PAD**	**No PAD**	**PAD**	**No PAD**
	**n = 743**	**n = 57, 062**	**n = 777**	**n = 74,223**
*Age* (*Mean* ± *SD*)	58.1 ± 13.0*	46.3 ± 15.8	62.4 ± 11.3*	48.0 ± 16.5
*Male*	47.24%	48.62%	59.46%*	48.10%
*College*/*University education*	36.07%*	44.64%	28.19%*	41.22%
*Currently employed*	31.09%*	56.92%	21.88%*	55.48%
*Body mass index category*				
Underweight	2.02%	2.81%	1.42%	1.80%
Normal	29.21%*	42.32%	19.43%*	30.90%
Overweight	36.20%	33.17%	29.60%	32.30%
Obese	30.96%*	19.01%	48.52%*	32.67%
Decline to answer weight	1.62%	2.69%	1.03%*	2.32%
*Currently smoke*	33.92%*	28.35%	35.65%*	18.91%
*Currently drink*	69.31%*	78.84%	51.61%*	65.20%
*Regularly exercise*	46.43%*	57.01%	39.51%*	65.30%
*Charlson comorbidity index* (*Mean*, *SD*)	1.8*	0.72	2.69*	0.92
*Comorbidity clusters*				
Cancer	11.84%*	4.93%	20.33%*	8.72%
Gastrointestinal	44.15%*	23.32%	51.74%*	23.23%
Infectious disease	4.98%*	1.51%	7.08%*	1.76%
Arthritis	20.73%*	8.45%	54.83%*	19.42%
Psychiatric	36.61%*	18.58%	44.27%*	21.83%
Respiratory	34.72%*	21.97%	53.15%*	31.43%
Renal	5.38%*	1.07%	7.85%*	0.87%
Liver disease	1.21%*	0.16%	1.42%*	0.19%
Diabetes	20.19%*	6.12%	38.35%*	10.51%
Cardiovascular	73.89%*	35.78%	88.16%*	42.28%
Previous MI	10.90%*	1.65%	26.25%*	2.82%
Cerebrovascular	8.21%*	1.47%	15.06%*	2.12%

*PAD and no PAD groups differ within region, p < .05.

**Figure 1:**
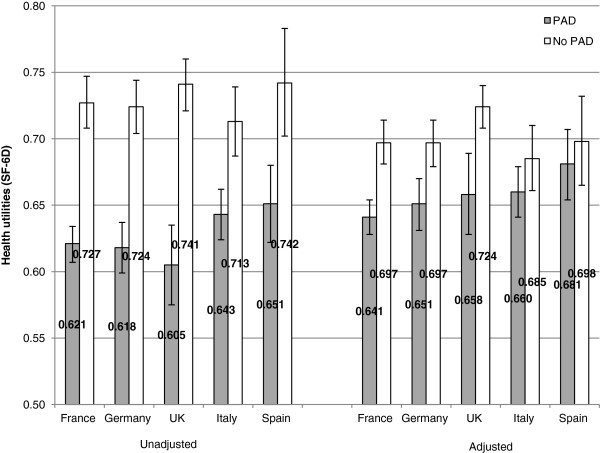
Unadjusted and adjusted health state utility differences between those with and without PAD by 5EU country.

Health outcomes of interest (i.e., health-related quality of life, work productivity, activity impairment, and healthcare resource use), were also similar between the two regions. Patients with PAD in the 5EU and the US reported significantly lower health utilities and lower mental and physical component summary scores compared with patients without PAD (all p < .05) (Table 2). These differences remained significant in adjusted analyses (Table 3). Given its importance as a single indicator of health status, health utilities were also examined by individual country in 5EU (Figure 1). Within each country, those with PAD reported significantly lower mean health utilities than those without PAD; the unadjusted decrease in utilities, i.e., the disutility ranged from 0.07 to 0.13. The adjusted disutility ranged from 0.02 to 0.06.

**Table 2:** Unadjusted mean patient-reported outcomes of those with and without PAD in the 5EU and United States

	**5EU**					
	**PAD**		**No PAD (Age matched)**		**No PAD**	
	**n = 743**		**n = 743**		**n = 57, 062**	
	**Mean**	**SD**	**Mean**	**SD**	**Mean**	**SD**
Mental component summary (MCS) score	43.44*	11.37	48.64	10.42	46.58	10.58
Physical component summary (PCS) score	37.57*	11.16	46.43	10.74	48.75	9.66
Health state utilities (SF-6D)	0.63*	0.12	0.73	0.14	0.73	0.13
Absenteeism %	12.08*	27.68	3.65	15.16	5.44	19.16
Presenteeism %	30.05*	27.42	13.77	21.03	15.84	22.8
Overall work impairment %	36.63*	33.33	16.26	24.73	19.39	27.69
Activity impairment %	46.54*	30.05	25.52	29.24	24.24	28.11
Number of physician visits	11.89*	13.66	5.66	6.24	5.34	7.37
Number of ER visits	0.40*	1.57	0.17	1.01	0.19	1.07
Number of hospitalizations	0.39*	1.54	0.12	0.62	0.13	1.16
	**US**					
	**PAD**		**No PAD (Age matched)**		**No PAD**	
	**n = 777**		**n = 777**		**n = 74,223**	
	**Mean**	**SD**	**Mean**	**SD**	**Mean**	**SD**
Mental component summary (MCS) score	45.51*	12.87	51.11	10	48.36	10.93
Physical component summary (PCS) score	31.77*	11.22	45.5	11.48	48.53	10.53
Health state utilities (SF-6D)	0.62*	0.14	0.76	0.14	0.75	0.14
Absenteeism %	12.80*	25.5	3.36	13.46	3.21	12.97
Presenteeism %	35.22*	30.46	13.99	22.95	14.37	22.63
Overall work impairment %	39.94*	34.34	15.62	25.3	16.12	25.11
Activity impairment %	56.67*	30.71	26.82	30.18	23.27	28.74
Number of physician visits	9.08*	9.63	4.98	6.61	3.86	5.87
Number of ER visits	0.65*	2.09	0.17	0.61	0.19	0.91
Number of hospitalizations	0.54*	1.56	0.12	0.44	0.11	0.91

*PAD group means differ from no PAD (age matched) group and no PAD group within region, p < .05.

Absenteeism, presenteeism, and overall work impairment were only asked among those who were currently employed.

**Table 3:** Adjusted mean patient-reported outcomes of those with and without PAD in the 5EU and United States

	**5EU**		**US**	
	**PAD**	**No PAD**	**PAD**	**No PAD**
	**n = 743**	**n = 743**	**n = 777**	**n = 777**
	**Adjusted mean**	**Adjusted mean**	**Adjusted mean**	**Adjusted mean**
Mental component summary (MCS) score	45.14*	46.95	47.71*	48.91
Physical component summary (PCS) score	39.77*	44.23	35.10*	42.18
Health state utilities (SF-6D)	0.66*	0.70	0.66*	0.72
Absenteeism %	7.68*	3.39	6.05*	1.58
Presenteeism %	23.37*	14.23	18.35	13.46
Overall work impairment %	28.68*	16.83	23.89*	14.26
Activity impairment %	38.27*	27.48	41.88*	29.20
Number of physician visits	9.31*	6.18	6.79*	5.49
Number of ER visits	0.24	0.16	0.35*	0.14
Number of hospitalizations	0.25*	0.11	0.37*	0.20

*PAD and no PAD groups differ within region, p < .05.

Adjusted means represent the predicted value for the outcome (separately for patients with PAD and age-matched patients without PAD), assuming all covariates are set at the sample mean. Covariates included gender, education level, BMI, smoking, drinking and exercise status, and comorbid conditions.

Absenteeism, presenteeism, and overall work impairment were only asked among those who were currently employed.

In both the 5EU and US, among those participants who reported being employed, those with PAD (EU n = 231; US n = 170) reported significantly greater levels of absenteeism, presenteeism, overall work impairment and impairment in daily activities than participants without PAD (EU n = 32,480; US n = 41,179) (all p < .05) (Table 2). Likewise, the number of physician visits, hospitalizations and number of emergency room visits in the past six months were greater for those reporting PAD versus those without PAD in both regions (all p < .05).

In analyses adjusted for participant demographics and health characteristics, all of the above-mentioned differences in health outcomes remained significant with the exception of the number of emergency room visits (5EU only), and presenteeism (US only). However, there was a trend for participants with PAD to have utilized more emergency room visits in the EU and to have a higher rate of presenteeism in the US when compared to their respective counterparts with no PAD (Table 3).

## Discussion

The objective of the current study was to estimate the burden of PAD across a wide array of health outcomes in the 5EU and the US. Although a number of studies have documented both the health-related quality of life effects of PAD along with its direct costs [12-14], these studies have often failed to adjust for demographics and health characteristics, making it difficult to assess the effect of PAD on patient related health outcomes. Further, many of these studies utilized convenience samples and it remains unclear what the overall PAD burden would be using a data source that is nationally representative with regard to age and gender. Finally, although direct costs have been previously studied, the extent to which PAD affects indirect costs through lost productivity has not been previously investigated. In this study of 5EU and US patients, those who reported being diagnosed with PAD reported significantly worse health-related quality of life, greater work and activity impairment, and greater healthcare resource use compared to those without PAD, and these differences remained after adjusting for participant demographics and health characteristics.

Consistent with prior research, our results reported a substantial health-related quality of life decrement among those diagnosed with PAD. Indeed, our adjusted physical component summary score mean for those with PAD in the 5EU (39.77) was over one standard deviation below the population mean of 50, consistent with what has been reported in previous studies [13]. Across both regions, the adjusted incremental differences between patients with and without PAD are also noteworthy as both physical component summary score and health utility means exceeded cutoff values for what could be considered clinically-relevant differences (3 and 0.03 points, respectively, based on pooled estimates from various therapeutic areas) [26,30]. This finding highlights the extent to which PAD can exert a clinically-meaningful effect on even generic assessments of health status.

Given the mean age of patients affected by PAD (58 years for the 5EU and 62 years for the US), it is not surprising that less than a third were still active in the labor force. Nevertheless, as suggested by prior research [17], patients with PAD reported significantly more work-related impairments than those without PAD, even after accounting for other demographic and health characteristics. In the adjusted analysis, approximately 25% of work time was missed either due to health-related absences or reduced productivity because of health (i.e., overall work impairment).

Although direct cost analyses were not part of the current study, the number of patient-reported resource utilization events was assessed. Past literature has suggested that the majority of PAD-related direct costs are due to the increased rate of hospitalizations [14], however, we also found a greater number of physician visits in the past six months for patients in the 5EU.

The results in the US were similar to those in Europe with respect to directionality and statistical significance. As with prior research [13], the mean adjusted physical component summary score (35.10) was more than one standard deviation below the population norm of 50. Work impairment was generally worse for those with PAD (with the exception of presenteeism), and the size of these adjusted effects was consistent with what was observed in the 5EU. Despite the older patient population, these results indicate that PAD is associated with a substantial increase in indirect costs. In the adjusted analysis, ER visits and hospitalizations were more frequent in the US than in the 5EU in absolute numbers, perhaps reflecting healthcare system differences. However, on a relative level (the percentage increase difference between patients with PAD versus controls) the effects were comparable between the regions.

### Limitations

Several limitations of this study should be noted. All data were self-reported including diagnosis of PAD and other comorbid conditions. Given the relatively high number of patients who remain undiagnosed with PAD, it is possible that some degree of misclassification may have occurred. As a result, the differences between groups may have been underestimated, though future research using more objective means of classifying patients would be necessary to determine the extent of any underestimation observed here. Work productivity and resource use variables were also self-reported and may have been subject to recall biases and other forms of measurement error. Because the SF-12v2 was used as the measure of health-related quality of life, only UK-specific utility weights were available to convert item scores to health utilities. Health utilities assessed with another instrument with country-specific weighting (e.g., EuroQoL-5D), might produce different effects. Although an attempt was made to rule out alternative explanations (demographics, health behaviors, comorbidities), it is possible that unmeasured variables might further explain the association between PAD and health-related outcomes. 5EU countries were pooled for analyses of work productivity and healthcare resource for the sake of sample size and brevity, yet this may have hidden underlying heterogeneity across these countries. The NHWS is representative of each country’s total adult population with respect to age and gender (and race/ethnicity in the US) but the PAD specific sample used here may differ in ways other than demographics from the PAD population which could influence the generalizability of the findings. For example, those participating in the NHWS may be higher functioning (as they are able to complete a survey) and therefore their outcomes might be underestimating the true effect of PAD. Additional studies should explore these research questions in other countries, to determine the universality of the observed effects.

## Conclusions

Collectively, these results suggest that PAD imposes a significant burden on patients in both the 5EU and the US. Patients with PAD exhibited poorer mental and physical health-related quality of life, greater healthcare resource use, and greater impairment in work and daily activities. Improved management of PAD may improve patient health-related quality of life and reduce the economic impact from a societal perspective.

## Abbreviations

PAD: Peripheral arterial disease; US: United States; SF-36: Short form-36; PCS: Physical component summary; MI: Myocardial infarction; EU: European union; 5EU: France, Germany, Italy, Spain, and the UK; NHWS: National health and wellness survey; CCI: Charlson comorbidity index; SF-12v2: Short form-12 version 2; MCS: Mental component summary; SF-6D: Short form-6D; WPAI: Work productivity and activity impairment; ER: Emergency room; BMI: Body mass index.

## Competing interests

This research was funded by Merck, Sharp and Dohme Corp. Dr. DiBonaventura is an employee of Kantar Health; Ms. Marrett and Dr. Zhang are employees of Merck and own stock in the company.

## Authors’ contributions

EM and QZ conceived the study and participated in the design and interpretation of the results and drafted parts of the manuscript. MD participated in the design, led the data analysis, participated in the interpretation of results, and drafted the manuscript. All authors read and approved the final manuscript.

## Supplementary Material

Additional file 1 Definition of comorbidity clusters. Click here for file
