# Supplementary material for: Burden of peripheral arterial disease in Europe and the United States: a patient survey
Source: Health Qual Life Outcomes. 2013 Oct 22;11:175. doi: 10.1186/1477-7525-11-175 (PMC3854518; doi:10.1186/1477-7525-11-175)
Supplement: Additional file 1 — Definition of comorbidity clusters. [file 1477-7525-11-175-S1.pdf]

Additional File 1: Definition of comorbidity clusters

**Cancer**: breast cancer, cervical cancer, colorectal cancer, leukemia, lymphoma, melanoma, metastatic solid tumor, non-small cell lung cancer, prostate cancer, small cell lung cancer, skin cancer, uterine cancer, and other cancer

**Gastrointestinal**: gastroesophageal reflux disease, heartburn, abdominal pain, abdominal bloating, irritable bowel syndrome, Crohn's disease, ulcerative colitis, ulcer disease, ulcers (active stomach or duodenal), and frequent diarrhea

**Infectious**: hepatitis B, hepatitis C, HIV, AIDS,

**Arthritis**: rheumatoid arthritis, osteoarthritis

**Psychiatric**: depression, anxiety, generalized anxiety disorder, panic disorder, social anxietydisorder, post-traumatic stress disorder, bipolar disorder, phobias, obsessive compulsive disorder, and attention deficit disorder

**Respiratory**: asthma, chronic bronchitis, emphysema, chronic obstructive pulmonary disease, allergies, hay fever, and pulmonary embolism

**Renal disease**: chronic kidney disease, moderate or severe renal disease

**Liver disease**: cirrhosis

**Diabetes**

**Cardiovascular disease**: hypertension, hyperlipidemia, angina pectoris, arrhythmia, congestive heart failure, and atherosclerosis

**Cerebrovascular disease**: stroke or mini-stroke/transient ischemic attack
